# Missing Data Statistics Provide Causal Insights into Data Loss in Diabetes Health Monitoring by Wearable Sensors

**DOI:** 10.3390/s24051526

**Published:** 2024-02-27

**Authors:** Carlijn I. R. Braem, Utku S. Yavuz, Hermie J. Hermens, Peter H. Veltink

**Affiliations:** Department of Biomedical Signals and Systems, University of Twente, 7522 NB Enschede, The Netherlands; s.u.yavuz@utwente.nl (U.S.Y.);

**Keywords:** missing data, health monitoring, signal processing, wearable sensors, biomedical sensors, continuous glucose monitoring, activity trackers, heart rate, vital signs

## Abstract

Background: Data loss in wearable sensors is an inevitable problem that leads to misrepresentation during diabetes health monitoring. We systematically investigated missing wearable sensors data to get causal insight into the mechanisms leading to missing data. Methods: Two-week-long data from a continuous glucose monitor and a Fitbit activity tracker recording heart rate (HR) and step count in free-living patients with type 2 diabetes mellitus were used. The gap size distribution was fitted with a Planck distribution to test for missing not at random (MNAR) and a difference between distributions was tested with a Chi-squared test. Significant missing data dispersion over time was tested with the Kruskal–Wallis test and Dunn post hoc analysis. Results: Data from 77 subjects resulted in 73 cleaned glucose, 70 HR and 68 step count recordings. The glucose gap sizes followed a Planck distribution. HR and step count gap frequency differed significantly (*p* < 0.001), and the missing data were therefore MNAR. In glucose, more missing data were found in the night (23:00–01:00), and in step count, more at measurement days 6 and 7 (*p* < 0.001). In both cases, missing data were caused by insufficient frequency of data synchronization. Conclusions: Our novel approach of investigating missing data statistics revealed the mechanisms for missing data in Fitbit and CGM data.

## 1. Introduction

Wearable sensors, such as fitness trackers and patches, enable continuous monitoring of physiological signals in free-living individuals. Currently, there are a wide variety of sensors to monitor health-related signals such as step count, heart rate, blood glucose level, blood pressure, respiratory rate, galvanic skin conductance and many others. Insight into this data can help individuals manage their health status and can empower patients in self-management of their chronic diseases, like diabetes, obesity and cardiovascular disease, especially combined with behavior change techniques [1,2]. For patients with diabetes, the continuous monitoring of glucose is vital to maintain glycemic control [3], whereas activity trackers provide detailed records of physical exercise for self-monitoring [4]. Overall, self-monitoring with wearable sensors could improve the overall quality of life and reduce health care costs by the prevention of comorbidities related to chronic diseases [1].

To accurately predict the health status of individuals, they must be monitored for an extended time. Monitoring time can be multiple days for physical activity [5,6] or two weeks when measuring blood glucose [7,8], or even multiple months when monitoring behavior change [9]. However, such longitudinal health monitoring will inevitably lead to data loss, caused by the device or sensor [10], actions by the subjects or a combination of the device and human interaction [11]. Most of the human-associated causes discussed in previous studies are based on participant adherence [12] or maintenance of devices (e.g., charging or synchronizing with storage device) [13]. In all cases, the missing data need to be addressed during data analysis, regardless of how they went missing.

Missing data in health monitoring pose one of the main challenges for signal processing, as missing data hinder real-time health status monitoring and most statistical and machine learning models cannot be directly applied [14,15,16,17,18,19]. Sometimes, the subject or parts of the data are not included in the analysis [20,21], but this leads to a reduction in sample size and decreased performances of classification algorithms [22,23,24]. Previous research in activity monitoring showed that missing data are hard to detect as both inactivity and missing data result in the same sensor output. As a result, deleting data in activity monitoring leads to biased estimates of physical activity and sedentary time [24,25,26]. Also, the definition of missing data for HR varies between studies [19,27]. Akturk et al. showed in glucose monitoring that missing data calls for longer monitoring periods to accurately reflect glycemic control [28]. At the same time, missing data can in some cases be informative and indicative for the health status [29].

Rubin classifies missing data in time-independent data based on the underlying mechanism [30]. Similar classifications are also applicable in times series [31,32]. Missing completely at random (MCAR) are missing data which are not systematically missing. In this case, the observed missing data are independent of other variables and the observed value itself. When the observed missing data are dependent on time and not on the observed value, they are missing at random (MAR). Finally, missing not at random (MNAR) occurs when missing data are related to time and the observed value. All three types of missing data types can be present in long-term health monitoring [13]. Knowledge of the underlying missing data characteristics and statistics can be used to select an appropriate imputation algorithm or design a missing data simulator to verify imputation algorithms [31,32]. In addition, with a structured and detailed analysis of the missing data characteristics in time, inferences about data loss mechanisms could be made.

The statistical characteristics of missing data in time are especially useful to deduce the mechanism of missing the data. In time series, gaps occur which are consecutive missing data points. The frequency of each gap size can be represented in a gap size probability distribution. When this distribution follows an exponential decline, the missing data are likely to be M(C)AR [31,32]. Moreover, missing data in time series are closely related to behavior in time. Especially in health monitoring, missing data in time are linked to human behavior by, for example, the circadian rhythm, week patterns, and other daily-life patterns [13,15]. Both Lee and also Lee and Gill showed that missing data in activity monitors are related to the time of day [24,33]. In this case, missing data are not evenly dispersed over time and they are MAR instead of MCAR. Unequal missing data dispersion is likely to influence research outcomes. For example, in glucose monitoring, the type of data loss affects the predictive value more than the amount of missing data [28]. Missing data patterns also affect the performance of imputation algorithms, as larger gaps and MNAR data are more difficult to impute [14,33]. On the other hand, other research showed that missing data patterns can be an informative missingness which can be used for clustering [34].

Although other papers have investigated missing data statistics, we hypothesize that more information can be inferred from a thorough evaluation of missing data statistics in continuous health monitoring data. Therefore, this study aimed to systematically investigate gap size probability distribution and missing data dispersion. For this, we introduced a novel data analysis method to determine the missing data mechanism. In this paper, we use data from a glucose monitor and an activity tracker used in type 2 diabetes patients. These data give a real-world reflection of health monitoring, as the activity tracker is commonly used for long-term health monitoring in a variety of individuals, whereas the CGM is an important monitoring device in patients with diabetes.

## 2. Materials and Methods

### 2.1. Data Set

Wearable sensor data from the DIAbetes and LifEstyle Cohort Twente (DIALECT) were used in this study. DIALECT is an observational study in patients with type 2 diabetes who are treated in the Ziekenhuis Groep Twente hospital in Almelo and Hengelo in The Netherlands [35]. Those eligible for participation were adult patients with type 2 diabetes from the hospital’s outpatient clinic. Exclusion criteria were end-stage kidney disease and inability to provide consent.

In a sub-cohort of DIALECT, a continuous glucose monitor (Freestyle Libre^®^, Abbott Diabetes Care, Alameda, CA, USA) and an activity tracker (Fitbit Charge HR or Charge 2A, Fitbit, San Francisco, CA, USA) were added for additional health monitoring (DIALECT-2) [36]. During the two-week study period, patients were instructed to maintain their usual activities, but needed to remove the activity tracker for bathing and swimming. Patients were allowed to remove the activity tracker during the night if the wristband was uncomfortable. The continuous glucose monitor (CGM) can be worn up to 14 days. It can store a maximum of 8 h of data, after which it needs to be manually synchronized with a receiver. In this paper, data collected from September 2017 to November 2019 were used. Next to the continuous data from the continuous glucose monitor and activity tracker, demographic data were collected. In this study, we used age, gender, body mass index, glycated hemoglobin (HbA1c), years since diabetes diagnosis and medication use for type 2 diabetes mellitus.

DIALECT-2 was performed in accordance with the Helsinki agreement and guidelines of good clinical practice. This study was approved by the local institutional review boards (METC-registration number NL1009.68020). All patients signed an informed consent form before participation in this study.

### 2.2. Data Pre-Processing

**Figure 1:**
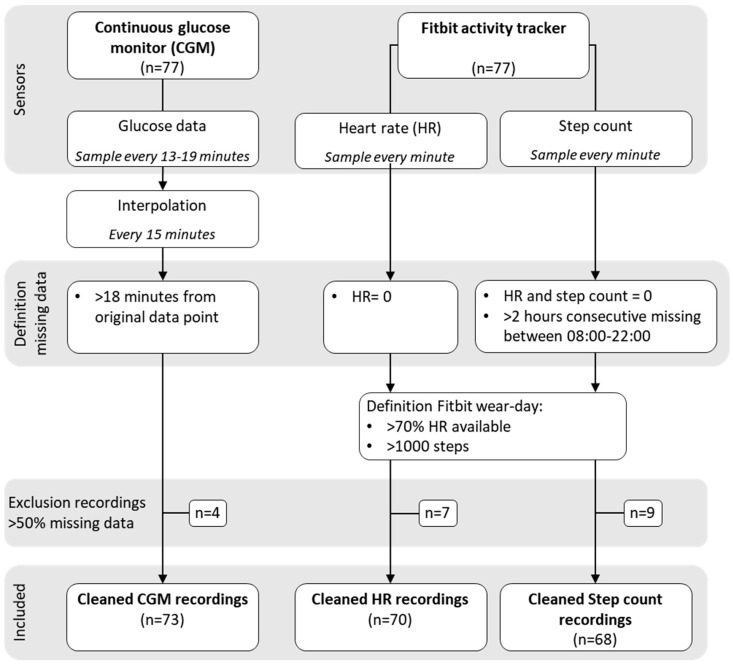
Overview of the data processing steps.

The data of the sensors were processed individually. The missing data and exclusion criteria were defined for each sensor data. An overview of the processing steps can be found in Figure 1.

CGM data sampling was time-variant, with a median sampling rate of 15 min. Because of the need for time-invariant sampling in analysis, CGM data were resampled with a sampling rate of 15 min using linear interpolation. Data were regarded as missing when the interpolated sample was 18 min away from two original data points. As the sample interval was less than 19 min 99% of the time, all longer sampling intervals indicated missing data.

The activity tracker measures heart rate (HR) and step count data with a sampling frequency of 1 sample/minute. Unfortunately, there is currently no consensus on the definition of wear time in Fitbit sensors when evaluating physical activity [27]. The activity tracker produces a zero value when no measurement is available. Missing data therefore first needs to be defined every minute. Similar to previous studies assessing physical activity with Fitbit devices, we defined zero values in HR as missing [27,37]. When both HR and step count values were zero, we classified step count as missing [38]. Also, 2-h periods of no step count during 8:00–22:00 were regarded as missing data [39]. Hereafter, we excluded days with insufficient wear time. Most studies using Fitbit for physical activity monitoring only use data during the day. However, we extended these wear time criteria to a 24-h day. Most report that HR should be available for more than 10 waking hours [27,37,40,41]. Therefore, we chose that HR should be available 70% of the time in 24 h. Also, there should be more than 1000 steps recorded during the day for sufficient wear [27,40,42]. Any glucose, HR or step count recording with more than 50% data loss was excluded.

### 2.3. Data Analysis

After pre-processing of the data, the recording length and percentage of missing data were calculated for data of all sensor modalities based on the criteria introduced in the previous section. After that, to test whether missing data were correlated between the sensor modalities, the Spearman correlation was examined between the percentage of data loss in glucose, HR and step count data for each individual [43]. Later, data loss characteristics in these three types of data were examined.

### 2.4. Statistics of Missing Data and Missing Mechanisms

**Figure 2:**
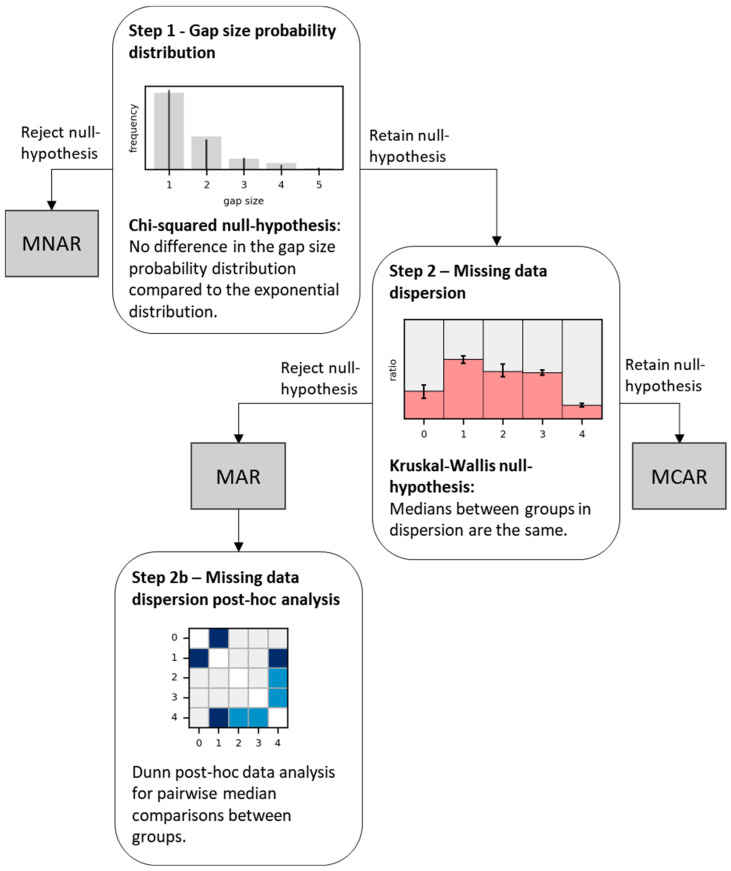
Overview of the tests to determine the missing data mechanism in continuous data using missing data statistics. MAR = missing at random, MCAR = missing completely at random, MNAR = missing not at random.

Investigating missing data characteristics can reveal the missing data mechanism (Figure 2). For this, we first classified missing data based on their statistical distribution as described by Zhou et al. [32]. This classification uses the properties of Poisson distribution to describe MCAR. A Poisson process is a random process where the intervals’ probability of events have an exponential distribution [44]. When the gap size probability distribution over the whole recording follows an exponential decline, the missing data process can be considered MCAR. When the gap size probability distribution differs significantly from an exponential function, the missing data mechanism is not a Poisson process and is assumed to be MNAR. The difference between an exponential probability mass function and the gap size probability is determined with the Chi-square test (Step 1).

If the missing data have a time dependence to a certain extent, the process is not completely random and is classified as MAR [31,32]. We estimated the missing data dispersion (the probability of observing missing data in a period, for example, during the day) to understand the missing data pattern over time. These results determined whether missing data were significantly dependent on time and therefore MAR (Step 2).

#### 2.4.1. Step 1—Gap Size Probability Distribution

To determine whether missing data are MNAR, the gap frequency was calculated for glucose, HR and step count data pooled over all subjects. For this, the size and occurrence of the gaps were determined from the recordings and summated. Thereafter, the gap frequency was fitted with a maximum-likelihood estimation of the Planck probability mass function (pmf), which is a normalized exponential pmf, as well as other probability distribution functions: beta-binomial, Boltzmann (truncated discrete exponential), Laplacian, geometric, Poisson, logarithmic, negative-binomial, hyper-geometric, uniform, Skellam, Yule–Simon, Zeta, Zipf and Zipfian [45]. The Zipf(ian) pmf is derived from Zipf’s law, which states that the frequency of a gap is inversely proportional to its frequency rank and is often used to study natural linguistics and the distribution of city sizes. The goodness of fit between the gap frequency and the fitted Planck pmf was analyzed with the Chi-square test and the sum of squared errors (SSE). The Chi-square test is only valid for larger frequencies [46]. Therefore, we cut off at the first gap size with a frequency equal or smaller than five. We assumed the missing data were MCAR when the gap frequency was significantly different from the fitted Planck pmf and the one-way Chi-square test was below the significance threshold.

#### 2.4.2. Step 2—Missing Data Dispersion

To test whether missing data were MAR or MCAR, the missing data dispersion was investigated. For this, the glucose, HR and step count data of all subjects were sorted into several groups in time: every hour of the day (00:00–01:00, etc.), weekdays (Monday through Sunday), business days (Monday through Friday) and weekends (Saturday to Sunday), and measurements days (day 1, day 2, etc., from the start of measurement). To determine the data loss dispersion, the percentage of missing data in each group was calculated for each individual. The percentage of missing data in each group was visualized with a bar graph with the median percentage of data and missing data and interquartile range for each group (IQR). The Kruskal–Wallis test and Dunn post hoc analysis with Bonferroni correction for multiple testing were used to test whether the data loss differed significantly in the grouped time periods. The Kruskal–Wallis test was used to determine whether there is a significant difference between the median data loss in all groups. To determine which groups’ medians were significantly different, the Dunn analysis was used. This analysis used the same data as the Kruskal–Wallis to test the differences between the groups. The ANOVA with Tukey post hoc was the parametric alternative of these tests. When the Dunn post hoc analysis revealed that missing data differed significantly for over time, we assumed the data were MAR. When no significant differences over time were found, the missing data were assumed to be MCAR.

### 2.5. Subgroup Analysis

To determine whether missing data were related to subject characteristics, subgroup analysis was performed. The subgroups were divided based on the amount of missing data and on descriptive data (Table 1).

#### 2.5.1. Subgroups Based on Missing Data

For subgroup analysis based on the amount of missing data, we divided the recordings based on the percentage of missing data into three groups (0–10%, 10–20%, >20%). We tested whether descriptive subject characteristics differed between these groups with the Kruskal–Wallis test and Dunn post hoc analysis for continuous variables or Chi-square test for dichotomous variables. The descriptive subject characteristics tested for were gender (male vs. female), age, BMI, Hb1Ac, years since DM2 diagnosis and the use of oral, insulin and other diabetes type 2-related medications. We applied a Bonferroni correction for multiple testing, both for the group test and post hoc analysis. For the three subgroups, the gap size probability distribution and data loss dispersion were studied and tested as described above. Additionally, we tested if the subgroup gap frequency was the same as for all subjects combined. The root-mean-square-error (RMSE) between the normalized medians of the missing data dispersions was calculated to compare the differences in missing data dispersions between the subgroups.

#### 2.5.2. Subgroups Based on Descriptive Data

We divided the recordings of glucose, HR and step count data based on the descriptive subject characteristics. The following subgroups were made: gender (male vs. female), age (<50, 50–60, 60–70, >70 years), normal weight and overweight (BMI < 30) and obese (BMI > 30 kg/m^2^ [47]), Hb1Ac clinical targets (≤53, >53 mmol/mol [48]), years since DM2 diagnosis (0–10, 10–20, >20) and the use of oral, insulin and other diabetes type 2-related medication. For these groups, we tested the differences in the percentages of missing data with the Kruskal–Wallis test and Dunn post hoc analysis and applied a Bonferroni correction for multiple testing. Thereafter, we determined the gap frequency and missing dispersion over time for all subgroups. Additionally, we tested if the subgroup gap frequency was the same as for all subjects combined. The root-mean-square-error (RMSE) between the normalized medians of the missing data dispersions was calculated to compare the differences in missing data dispersions between the subgroups.

### 2.6. Statistics

We report the median, IQR and range, e.g., for the descriptive subject characteristics, recording length and percentage of missing data. A *p*-value of 0.01 is considered significant.

## 3. Results

In DIALECT-2, 77 subjects had glucose data from a CGM and HR and step count data from a Fitbit activity tracker. Subject characteristics of the whole group can be found in Table 1.

### 3.1. Exclusion of Recordings

Multiple recordings were completely excluded from further analysis based on criteria described before (see Figure 1). From the glucose data, four recordings were deleted, as there was more than 50% data loss (55.3%, 67.8%, 77.9% and 78.9%). The median number of days not being classified in the Fitbit data was 3 (IQR 2–6, range 2–15). Seven HR and nine step count recordings were excluded, as they had more than 50% data loss. An overview of the recording length and missing data of the included recordings can be found in Table 2. There was a strong correlation (0.83) between the amounts of missing data for HR and step count (*p* < 0.001); no other correlation was significant.

### 3.2. Gap Frequency

**Figure 3:**
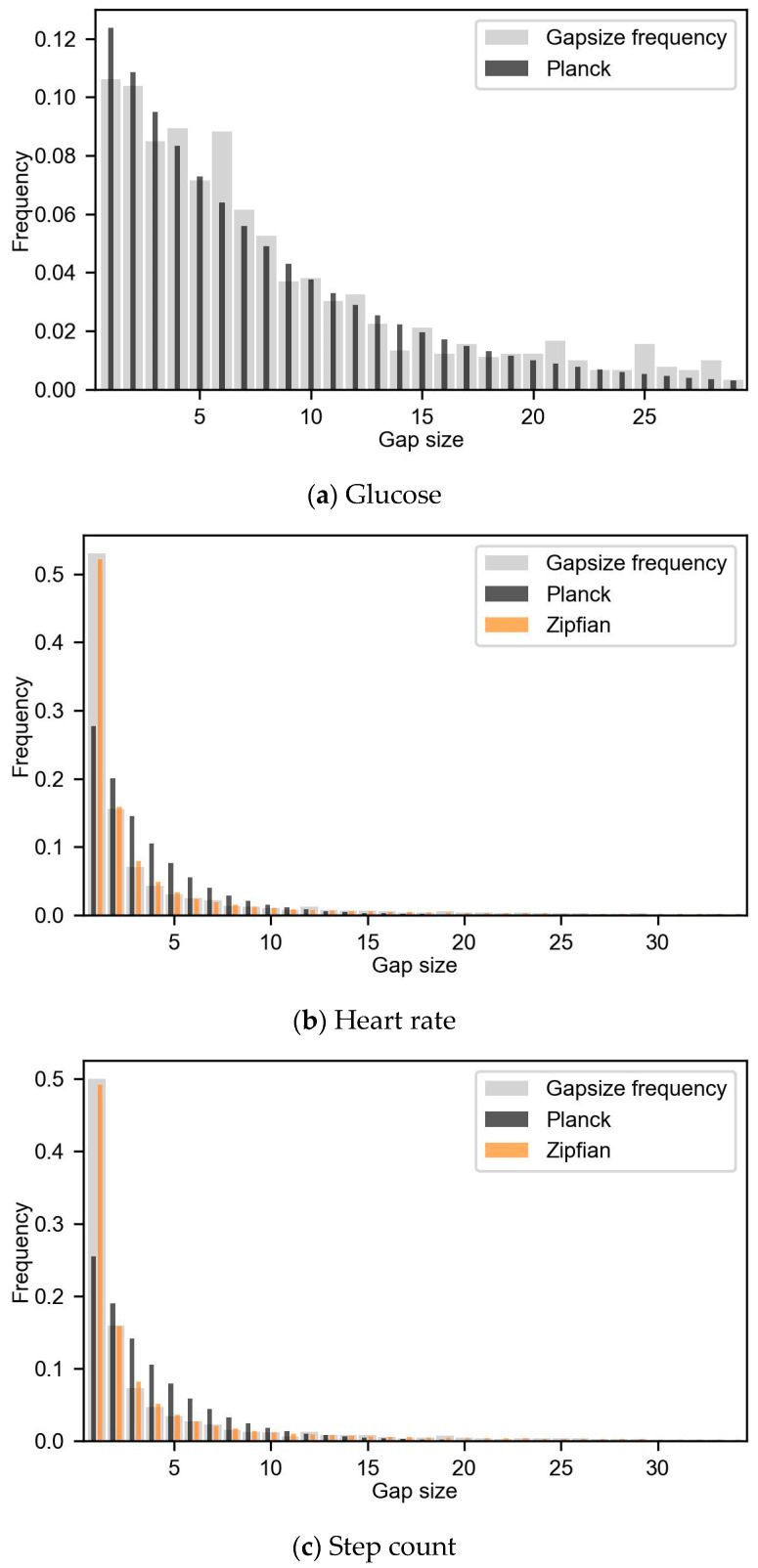
Gap size probability distribution (light grey) with the fitted Planck probability mass function (pmf) in dark grey for the three types of data. (**a**) Glucose gap frequency of glucose data with Planck pmf. (**b**) Heart rate gap frequency with fitted Planck and Zipf pmf in orange. (**c**) Step count gap frequency with Planck pmf, which starts at 16 samples due to the step count pre-processing.

The number of gaps summated over the recordings in glucose, HR and step count data were 97, 5682 and 4558, respectively. The gap frequency was cut off at the first gap size, which occurred less than five times. The gap size probability distribution was cut off at gap sizes of 29, 34 and 34 for glucose, HR and step count data, respectively. Figure 3 shows the gap frequency, Planck pmf and alternative pmf for glucose, HR and step count.

The glucose gap frequency followed the Planck pmf (Figure 3a) and was not significantly different therefrom (*p* > 0.99 and SSE of 1.5 × 10^−3^). So, glucose gap frequency followed an exponential decline and had M(C)AR missing data. The HR and step count gap frequency did not follow the Planck pmf (Figure 3b,c) and were in both cases significantly different (HR *p* < 0.001 and SSE of 8.0 × 10^−2^, and for step count *p* < 0.001 and SSE of 7.3 × 10^−2^). However, the Zipfian pmf had the best fit with HR and step count gap frequency with SSEs of 2.6 × 10^−6^ and 2.3 × 10^−4^, respectively. Consequently, the missing data in both HR and step count were MNAR.

### 3.3. Missing Dispersion

**Figure 4:**
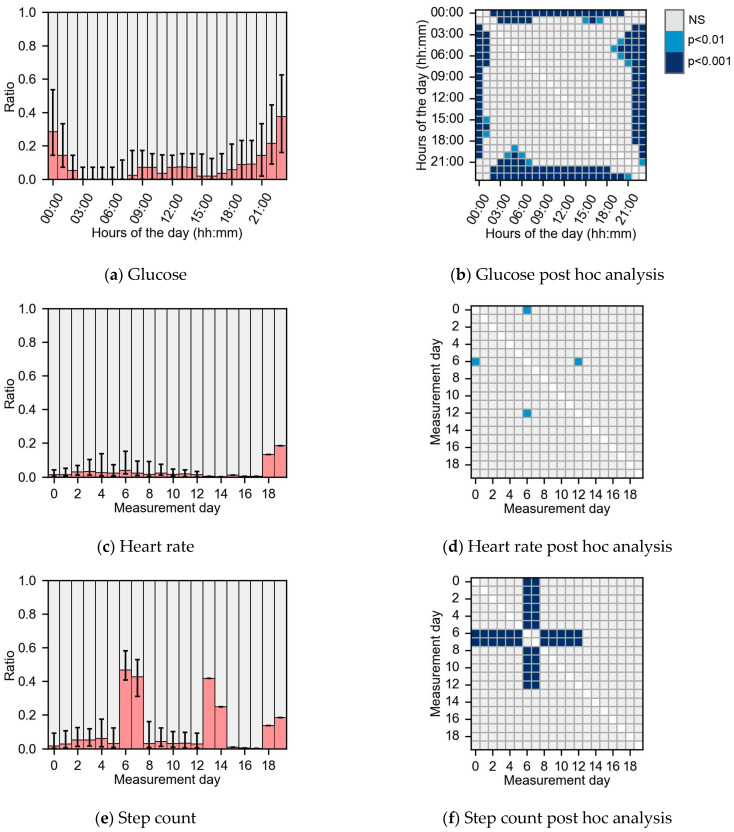
Three missing data dispersions over time in the left panels with (in red) the ratio of missing data. In the right panels, the subsequent Dunn post hoc analysis. (**a**) Missing data dispersion in glucose data in hours of the day and (**b**) post hoc analysis to test for differences in glucose data between hours of the day. (**c**) Missing data dispersion in heart rate data over measurement days and (**d**) post hoc analysis for test differences between heart rate measurement days. (**e**) Missing data dispersion in step count data over measurement day and (**f**) post hoc analysis for test differences between step count measurement days.

Examples of missing data dispersion are shown in Figure 4 for glucose, HR and step count data. Figure 4a depicts the missing data dispersion for glucose per hour of the day, with post hoc analysis in Figure 4b. The post hoc analysis showed that the amount of missing data between 22:00–01:00 was more than for most other hours during the day (*p* < 0.001). These missing data were caused by the storage capacity of 8 h for the CGM device in combination with subjects’ sleeping behavior. In addition, more missing data were found for measurement days 4, 5 and 7 than for the last measurement day 14 (*p* < 0.01). HR data had more missing data on measurement day 7 compared to days 1 and 13 (*p* < 0.01), as shown in Figure 4c,d. No other significant dispersion differences were found in the HR data.

Step count had significantly more missing data for measurement days 6 and 7 than for all other days (*p* < 0.001, Figure 4e,f). This peak in missing step count data was caused by the storage capacity of the activity tracker, which stopped recording step count when the data storage was full. All subjects had missing data for measurement days 6 and 7, which influenced other dispersion analyses. As subjects started with measurements always on Thursdays, there were significantly more missing data during business days compared to weekends (*p* < 0.001) and more missing data on Wednesdays and Thursdays compared to other days of the week (*p* < 0.001). We repeated the step count dispersion analysis excluding measurement days 6 and 7 and no other significant dispersions were observed.

### 3.4. Subgroups Analysis

To determine whether missing data might be related to descriptive subject data, subgroup analysis was performed. The subgroups were divided based on the amount of missing data and based on descriptive subject characteristics (Table 1).

#### 3.4.1. Subgroups Based on Missing Data

The subjects were grouped based on the amount of missing data (<10%, 10–20% and >20% missing data). The number of subjects per group for glucose, HR and step count data are presented in Table 3. We found no significant differences in the descriptive characteristics (Table 1) between the missing data groups of the glucose, HR and step count data groups.

**Figure 5:**
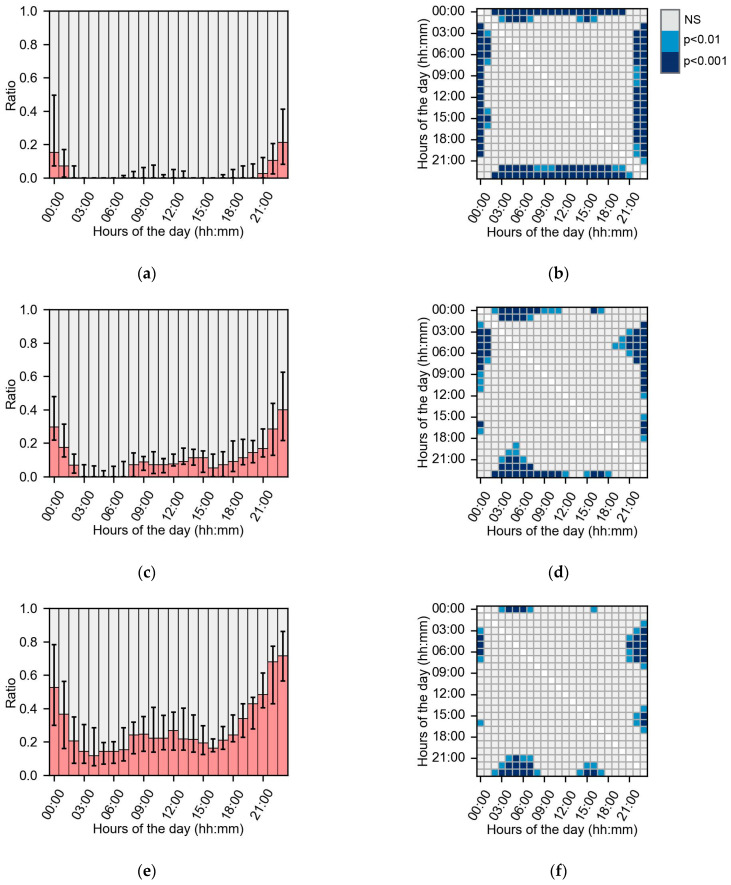
The missing data dispersion (left panels) and corresponding post hoc analysis (right panels) of missing data for every hour of the day in missing data subgroups. In (**a**,**c**,**e**), the red bars indicate the median ratio of missing data, and the error bars indicate the IQR. (**a**) Missing dispersion for hour of the day in group <10% missing data in glucose data. (**b**) Post hoc analysis for hour of the day in group <10% missing data in glucose data. (**c**) Missing dispersion for hour of the day in group 10–20% missing data in glucose data. (**d**) Post hoc analysis for hour of the day in group 10–20% missing data in glucose data. (**e**) Missing dispersion for hour of the day in group >20% missing data in glucose data. (**f**) Post hoc analysis for hour of the day in group >20% missing data in glucose data.

The gap frequencies were compared with the Planck pmf for glucose and Zipfian pmf for HR and step count data. None of the subgroups’ gap frequencies were significantly different from the tested distributions. Figure 5 shows the missing data dispersion of the glucose over hours per day for the three subgroups based on missing data. Figure 5a,c,e show the overall shapes of the missing data dispersion. The missing data dispersion between hours of the day in glucose data was different between <10 and >20% data loss, as the RMSE between the subgroups was 0.094. The RMSEs between the subgroup 10–20% data loss and subgroups <10% and >20% data loss were lower, 0.043 and 0.059, respectively.

#### 3.4.2. Subgroups Based on Descriptive Data

To see whether descriptive data can predict missing data, the recordings were grouped based on the descriptive data (Table 1). However, there was no significant difference in the amount of missing data found for the subgroups based on the descriptive characteristics.

**Figure 6:**
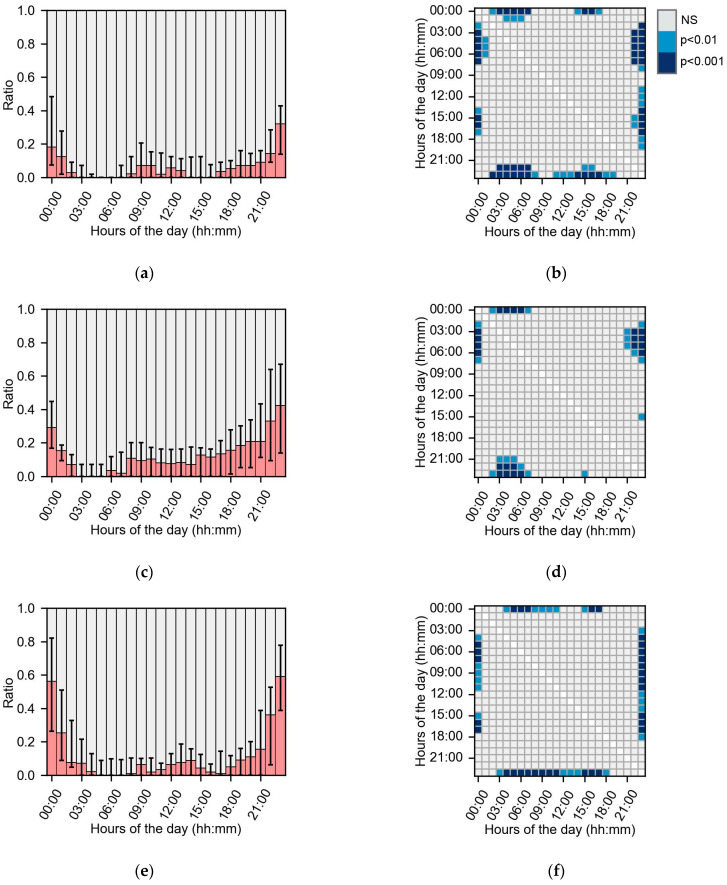
Missing data distribution (left panels) in glucose data in the subgroups based on years of type 2 diabetes diagnosis for every hour of the day and corresponding post hoc analysis (right panels). In (**a**,**c**,**e**), the red bars indicate the median ratio of missing data, and the error bars are the IQR. (**a**) Glucose missing data dispersion for every hour of the day in subgroup <10 years since diagnosis of type 2 diabetes (*n* = 33). (**b**) Post hoc analysis for hour of the day dispersion in <10 years since diagnosis of type 2 diabetes. (**c**) Glucose missing dispersion for every hour of the day in subgroup 10–20 years since diagnosis of type 2 diabetes (*n* = 25). (**d**) Post hoc analysis for hour of the day dispersion in 10–20 years since diagnosis of type 2 diabetes. (**e**) Glucose missing dispersion for every hour of the day in subgroup >20 years since diagnosis of type 2 diabetes (*n* = 16). (**f**) Post hoc analysis for hour of the day dispersion in >20 years since diagnosis of type 2 diabetes.

The subgroup gap frequencies were compared with the Planck pmf for glucose data and Zipf pmf for HR and step count data. None of the subgroups’ gap frequencies were significantly different. The glucose missing data dispersion over hours of the day for the subgroups <10, 10–20 and >20 years of type 2 diabetes diagnosis are shown in Figure 6. The figures show differences in the missing dispersion contour over time and in the post hoc analyses. Missing data were more concentrated around 23:00–01:00 in the group with >20 years diagnosis than in the other groups. The RMSEs for the missing data medians between subgroup >20 years and subgroups <10 years and 10–20 years of type 2 diagnosis were 0.103 and 0.086, respectively, whereas the RMSE between subgroups <10 years and 10–20 years diagnosis was only 0.057. In summary, missing dispersions in glucose data were influenced by the years of diagnosis.

## 4. Discussion

Missing data are unavoidable in health monitoring with wearable sensors. Missing data mechanisms can be uncovered with the novel analysis of the missing data statistics presented in this paper. In this paper, we applied this structured approach to look at missing data statistics in glucose, heart rate and step count data from wearable sensors in a population with type 2 diabetes. The HR and step count gap size probability distribution was MNAR as it was significantly different from an exponential decline. We hypothesized that the missing data in HR was MNAR resulting from device errors. Step count missing data were also MNAR, due to the close correlation with HR as a result of the pre-processing of the data. Significant differences over time in glucose data revealed that the missing data were MAR. The MAR data resulted from the erroneous human use of the device as the storage capacity became full over time and was not synchronized in time to prevent data loss. This structured analysis of the missing data allowed us to infer the mechanism behind missing data. The analysis of the gap size probability distribution and missing dispersion over time can be applied to other studies using time series data to understand the cause of missing data.

The use of the gap frequency and missing data dispersion allows for a detailed analysis of the missing data mechanisms. The gap frequency of HR and step count showed that there are more gaps of one sample than would be expected with an exponential decline. The step count missing patterns closely follow the HR missing patterns, and HR was used to determine when data were missing in step count. The reason for the increased amount of one sample gaps in HR cannot be attributed to human behavior, the research protocol or the pre-processing of the HR data. The increase was therefore likely caused by the monitoring device itself. One hypothesis might be that the HR sensor disconnected from the skin for short periods of time. The gap frequency in the glucose data followed an exponential decline, and thus was not considered MNAR. The missing data dispersion in the glucose and step count both had significant differences over time and were both caused by the storage capacities of the devices in combination with infrequent data synchronization of the devices. Significantly, more missing data were present at night than during the day for glucose data. This was a direct consequence of the storage capacity of the sensor. The on-skin sensor (Free-Style Libre 2) can store data on the patch for up to 8 h, whereafter the oldest data are deleted to make space for new data. Together with human sleeping behavior of around 8 h, this resulted in more missing data at the beginning of the night. Data dispersion analysis for step count showed significantly more missing data on measurement days 6 and 7, which again was caused by the storage capacity of the activity tracker. The storage capacity of the activity tracker was reported to be 7 days when recording HR and step count simultaneously. Therefore, the researchers synchronized the device in hospital at return visits after a week. However, in hindsight, the storage capacity of the activity sensor is only around 6 days while recording both HR and step count and this caused deletion of step count data when the storage of the activity tracker was full. These results suggest that the missing data in glucose and step count data were both MAR and missing data was caused by a combination of the device’s storage capacities and incorrect handling of the device during the study and could therefore be attributed to human misuse. Without a structured analysis of the missing data characteristics, this knowledge of the missing data mechanism would not have been uncovered. This way of analyzing missing data is likely to detect missing data on a group-based level, as the data of all research participants were pooled and individual habits were averaged out. Early evaluation of missing data during the study inclusion could show where more missing data are unexpectedly happening and these could therefore be reduced.

The subgroup analysis did not show any significant differences in the amount of missing data or descriptive characteristics. Another study did show that participant adherence using a fitness tracker is related to age, personality traits and early adherence to the protocol [49]. Our results show that the missing data dispersions over time were different between the low and high data loss subgroups (Figure 5a,e). In the low data loss group, missing data mostly occurred at night, whereas in the high data loss group missing data occurred throughout the day. Therefore, subjects in the high data loss group did not scan the sensor frequently enough and had lower adherence to the research protocol. Also, missing data dispersion was influenced by the years of type 2 diabetes diagnosis (Figure 6). The observed difference might be caused by the changes in sleep patterns, as type 2 diabetes is linked with disordered sleep [50]. Therefore, the variance in dispersion of missing data might be caused by co-morbidities of type 2 diabetes mellitus. In summary, missing data dispersion is influenced by descriptive subject characteristics and the amount of missing data and could have an impact on the research outcomes.

### 4.1. Limitations

This study provides a structured approach to determine the amount of missing data and their statistical characteristics. The approach was applied on two different sensors and three types of data: glucose, HR and step count. However, during the data pre-processing, we defined what is considered as missing data in this paper. This missing data definition had a profound impact on the location of missing data and consequently on the missing data statistics. Therefore, with an alternative definition of missing, the outcomes of this paper are likely to change. However, we believe the methods are robust enough that the outcomes would stay the same.

We found that the storage capacity in combination with human handling of the devices played a key role in the missing data statistics in this study. Newer versions of the sensors used in this paper are already on the market with more storage capacity and will result in a reduction of missing data. As a result, the amount of missing data and missing data patterns presented in this paper are device- and protocol-specific. It is likely that missing data caused by the device have obscured missing data patterns caused by participants. However, the patterns caused by the device were not uncovered without the presented data analysis.

We studied the correlation between the three data modalities through Spearman correlation. There was a significant correlation between the HR and step count missing data, as both come from the same wearable device and because of the dependence in the pre-processing of the data (Figure 1). Nevertheless, a significant correlation between sensors would not imply a causation of missing data [43].

### 4.2. Future Research

The differences in missing data patterns caused by descriptive data should be taken into account in the design of future wearable sensor studies. A pilot study could be used to first identify where in time missing data might be occurring. These results can then be used to implement preventative measures, to reduce the amount of missing data.

Future research should focus on understanding how the statistical characteristics of missing data can influence research outcomes and the accuracy of classification algorithms and data imputers. To study this, artificial missing data will need to be introduced in data, which emulate missing data statistics found in real-world data.

Finally, diverse types of sensors and research populations need to be studied to see how research subjects’ behavior influences missing data.

## 5. Conclusions

The analysis of missing data statistics reveals the mechanism behind missing data in health data from wearable sensors in the type 2 diabetes population. In this study, we identify that missing data have distinctive characteristics based on data loss mechanisms including the device, handling of the wearables and population characteristics. Missing data from the same wearable device but different sensors can have different missing data mechanisms. Subject characteristics can have an influence on where data are missing over time. The analysis of missing data characteristics can be useful to select appropriate data imputers and allows for careful interpretation of the research outcomes.

## Figures and Tables

**Table 1:** Descriptive characteristics of the 77 research subjects.

	Median or *n*	[IQR] or %
Male	46	60.0%
Age (years)	64	[56–71]
BMI (kg/m^2^)	33	[29.0–35.9]
17.5–25	4	5.2%
25–30	22	28.6%
30–40	45	58.4%
>40	7	9.1%
Hb1Ac (mmol/mol)	58	[52–64]
≤53	27	36.5%
>53	50	64.9%
Years since diagnosis (years)	14	[7–20]
Use of type 2 diabetes medication	74	100%
Oral	69	89.6%
Insulin	43	55.8%
Other	30	39.0%

**Table 2:** Description of the number of recordings (n), recording length and percentage of missing data in glucose, heart rate (HR) and step count data after deletion.

	*n*	Recording Length (Days, Hours: Minutes)			Missing Data (%)		
	(%)	Median	[IQR]	[Range]	Median	[IQR]	[Range]
Glucose	73 (95%)	13, 19:15	[11, 21:15–13, 21:15]	[6, 22:15–20, 17:00]	11.1	[6.2–17.6]	[0.2–47.2]
HR	70 (91%)	13, 00:00	[12, 00:00–13, 00:00]	[2, 00:00–20, 00:00]	9.4	[3.3–18.0]	[1.2–44.6]
Steps count	68 (88%)	12, 12:53	[11, 21:51–12, 15:16]	[1, 11:44–19, 15:04]	15.4	[12.5–22.4]	[6.8–42.1]

*n* = number of recordings, HR = heart rate.

**Table 3:** Number of recordings in the subgroups based on missing data.

	Recordings in Subgroups Based on Missing Data		
	<10%	10–20%	>20%
Glucose	34 (47%)	23 (32%)	16 (22%)
HR	37 (53%)	20 (29%)	13 (19%)
Step count	15 (22%)	32 (47%)	21 (31%)

HR = heart rate.

## Data Availability

No new data were created or analyzed in this study. The data used in this study are not publicly available as subject inclusion is still ongoing.
